# Investigating the RAS can be a fishy business: interdisciplinary opportunities using Zebrafish

**DOI:** 10.1042/CS20180721

**Published:** 2018-12-05

**Authors:** Scott Hoffmann, Linda Mullins, Charlotte Buckley, Sebastien Rider, John Mullins

**Affiliations:** University of Edinburgh/BHF Centre for Cardiovascular Science, Queen’s Medical Research Institute, The University of Edinburgh, 47, Little France Crescent, Edinburgh EH16 4TJ, U.K.

**Keywords:** optogenetics, Renin, SPIM, transgenics, Zebrafish

## Abstract

The renin–angiotensin system (RAS) is highly conserved, and components of the RAS are present in all vertebrates to some degree. Although the RAS has been studied since the discovery of renin, its biological role continues to broaden with the identification and characterization of new peptides. The evolutionarily distant zebrafish is a remarkable model for studying the kidney due to its genetic tractability and accessibility for *in vivo* imaging. The zebrafish pronephros is an especially useful kidney model due to its structural simplicity yet complex functionality, including capacity for glomerular and tubular filtration. Both the pronephros and mesonephros contain renin-expressing perivascular cells, which respond to RAS inhibition, making the zebrafish an excellent model for studying the RAS. This review summarizes the physiological and genetic tools currently available for studying the zebrafish kidney with regards to functionality of the RAS, using novel imaging techniques such as SPIM microscopy coupled with targeted single cell ablation and synthesis of vasoactive RAS peptides.

## Introduction

The existence of renin was first established at the end of the 19th century, based on the pressor action of renal extracts [1]; however, its site of synthesis and secretion within the juxtaglomerular apparatus (JGA) was only identified in the mid-twentieth century [2]. The RAS is an ancient system that exists to some extent in all vertebrates and was present long before mammalian evolution. In mammals, the renin–angiotensin system (RAS) is primarily responsible for blood pressure and osmotic regulation and, although the involvement of the RAS in hypertension was proposed over 60 years ago [3], new components continue to be discovered and the range of its biological roles is still being elucidated. Indeed renin-expressing cells are intimately involved with vascular development, renal repair and regeneration, haematopoietic tissues and immune responses, suggesting ancestral roles in multiple biological mechanisms [4].

By studying renin and RAS in the evolutionarily distant zebrafish, it is anticipated that our understanding of the roles of RAS and renin-expressing cells will be further advanced through *in vivo* experimentation, fundamental cell biology and drug screens. This review will focus on the tools available for investigating the RAS in zebrafish and assess the evidence for its functionality.

## The mammalian renin–angiotensin system

The RAS is the main regulator of salt and water balance in adult mammals. Renin is synthesized, almost exclusively, within specialized perivascular mural cells of the kidney called juxtaglomerular (JG) cells, which are located along the afferent arteriole proximal to the glomerulus and comprise a key anatomical and functional feature of the JGA [5,6]. The JG cells store active renin in densely packed granules and contribute, together with contractile smooth muscle cells, to the regulation of glomerular blood flow and blood pressure [7]. Renin is synthesized as a preprohormone and activated by the removal of 43-amino acids from the renin precursor, prorenin [8,9]. Although ‘inactive’ prorenin is constitutively secreted and accounts for approximately 80% of the circulating renin, active renin is secreted in a regulated manner in response to conditions that threaten fluid homeostasis, such as a decrease in sodium concentration or a drop in arterial blood pressure [6,10]. Renin activity is the rate limiting step in the RAS cascade. The renin substrate, angiotensinogen, is constitutively secreted by the liver, and plasma levels are remarkably stable [11,12]. Secreted renin cleaves the C-terminus of angiotensinogen to form the decapeptide angiotensin I (AngI). AngI is biologically inactive and acts as a precursor for the main effector of the system, angiotensin II (AngII). The conversion of AngI to AngII occurs through the cleavage of two amino acids from the C-terminus of AngI by angiotensin-converting enzyme (ACE) [10]. ACE is a membrane-bound peptidase, expressed throughout the body, most notably within the lungs, and has also been detected on vascular endothelial and renal proximal tubule cells [13]. ACE also recognizes bradykinin as a substrate, inactivating it and preventing its vasodilatory effects and is a pivotal link between the two regulatory systems. AngII is the main effector of the RAS, acting on the At1 and At2 receptors [14]. Most of the observed physiological actions of AngII, including vasoconstriction, increased cardiac contractility, increased sodium reabsorption through the renal tubules, and inhibitory actions on renin expression (although this has recently been contested [15]), are dependent on its action on the At1 receptor. AngII binding to the At1 receptor stimulates the production of aldosterone in the adrenal cortex, which acts on the principal cells of the kidney to increase sodium reabsorption. AngII also exerts effects on cell growth, proliferation and inflammatory responses [16]. The At2 receptor has broadly opposing functions to the At1 receptor, for example, decreasing cell proliferation and vasoconstriction, and is highly expressed during development, however, expression in some tissues, such as the heart, decreases postnatally [17]. In addition to the classical RAS, several additional bi-products and smaller peptides have been documented, which have only recently gained pharmacological significance [18]. These include angiotensin 1-7, formed by the ACE homologue, ACE2 [19,20]. Ang1–7 acts on the MAS receptor, inducing anti-inflammatory responses and vasodilation in contrast with the actions of AngII on the At1 receptor [20].

The complexity of the RAS system is such that many questions about its regulation remain. The role of the prorenin receptor has been contentious [21,22], but recent evidence suggests it may be involved in sodium and water handling in the principal cells [23] and acid handling in intercalated cells of the collecting duct [21]. An overactive RAS has been linked to hypertension and various cardiovascular diseases [14,24,25]. Pharmacological inhibitors targeting the RAS cascade, in particular renin inhibitors, ACE inhibitors and angiotensin receptor antagonists, have proved to be highly effective for the treatment of hypertension [26–28].

## Renin in development

Renin cell progenitors appear early in embryonic development in multiple tissues including skin, bone marrow, spleen and adrenal, prior to their appearance in the kidney [29–31]. Mammalian kidney organogenesis progresses via three different developmental stages, the pronephros, which is the first and simplest kidney form, the mesonephros and, finally, the metanephros [32,33]. During kidney development, renin cells, descended from Foxd1^+^ stromal cells [34], are distributed along the developing renal arterial tree, particularly at branch points and along nascent vessels [35]. As development progresses, renin expression becomes restricted to the afferent arteriole, eventually being limited to granulated JG cells; however, vascular smooth muscle cells can be recruited to re-express renin when homeostasis is threatened. The Notch pathway is intimately involved in the recruitment process since conditional deletion of the transcription factor RBP-J from renin-expressing cells dramatically reduces both renin levels and blood pressure [36]. Although various disease models exist, the kidney is a challenging organ to access for *in vivo* studies [37], and the involvement of renin in kidney repair and development remains to be fully elucidated.

## Zebrafish development and the RAS

*Ex utero* development and early onset of organogenesis allow the investigation of developing organ function in real time and *in vivo*. Zebrafish transparency during early development makes it a highly desirable model to study genetic manipulations [38,39]. Zebrafish kidney development shares many similarities with that of the mammalian kidney; however, unlike in mammals, the pronephros is a fully functional anatomical unit that is integral to ion reabsorption and blood filtration and, as such, provides an excellent model of early kidney development [40,41].

Friedman and Kaplan [42] demonstrated the pressor activity of crude kidney extracts from freshwater fish. Nishimura and Ogawa [43] reported the presence of granulated JG cells in teleosts, the presence of which is indicative of renin synthesis, processing and secretion. In addition to verifying the presence of granulated JG cells, they also showed that AngII increased blood pressure. In recent years, the zebrafish has emerged as a leading model organism for the study of both development and disease pathology. The zebrafish genome contains orthologues to approximately 70% of genes known to be causal in human diseases, and as such the zebrafish is a valuable model species contributing widely to our understanding of both gene function and disease aetiology [44,45]. Liang et al*.* [46] demonstrated the presence of a functional renin gene in zebrafish, the first such report in a non-mammalian species. Fournier et al*.* [47] utilized published gene and protein sequence databases to investigate the evolution of the RAS system and identified gene sequences orthologous for the majority of human RAS proteins, in multiple vertebrate species, the existence of which correlated with the presence of JG cells. The zebrafish was found to contain eight out of the nine sequences orthologous to the human genes: Ace1, Ace2, angiotensinogen, At1, At2, renin receptor, mineralocorticoid receptor and renin. No sequence was identified for the Mas receptor [47].

The renin–angiotensin system in teleost fish has been linked to the ability to survive in water of fluctuating osmolarity, which can lead to increased osmotic stress [48]. Zebrafish larvae in low salt conditions have increased renin expression and also higher concentrations of AngII, suggesting that RAS is involved in salt handling in this species [49–51]. The presence of At1 receptors in zebrafish was shown by Tucker et al*.* [52], with expression corresponding to tissues known to be involved in ion regulation. It is important to note that zebrafish also have additional mechanisms that allow them to maintain salt and water balance with their surrounding environment. The developing gills of larval fish are initially required for ion regulation rather than oxygen uptake [53,54], and larval zebrafish actively take up ions from the environment via specialised epithelial cells – ionocytes – on the integument [55].

## The zebrafish pronephros

**Figure 1:**
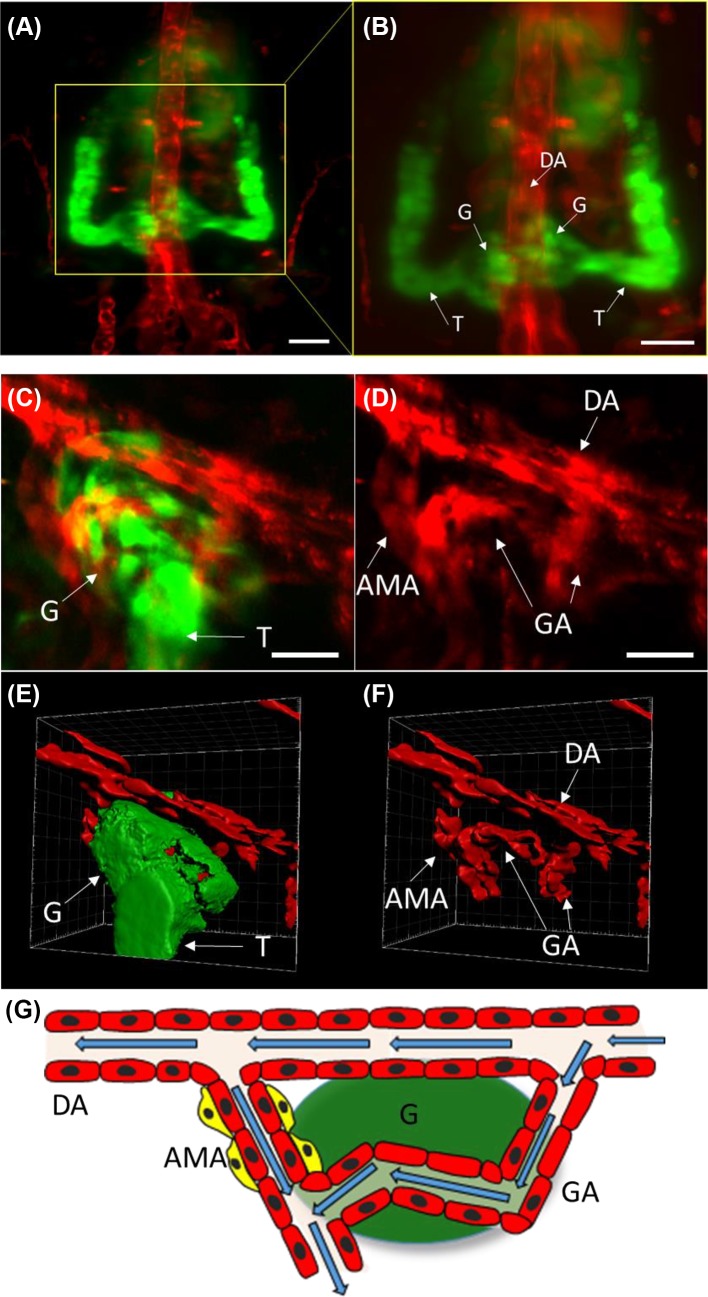
Interrelationship of Vasculature and nephron in early pronephric kidney (**A** and **B**) 3dpf live *Tg(wt1b:GFP;kdrl:mCherry*) larvae were anaesthetized, embedded in 1% agar and mounted on a Zeiss Z1 lightsheet microscope, head down and imaged dorsally. The dorsal aorta (DA) can be seen bisecting the image in red, with the pronephric glomerulus (G) in green fused at the midline and the pronephric tubules (T) draining from this. Images were acquired with at 20X/1.0 NA Water Plan Apo objective using dual beam illumination at 488 nm (*wt1b:GFP*) and 561 nm (*kdrl:mCherry*), and the images merged and presented as a maximum intensity projection. Scale bars represent 30 μm. (**C**–**G**) 5dpf live *Tg(α-sma:mCherry;wt1b:GFP*) larvae were anaesthetized, embedded in 0.5% agar and mounted on an LaVision TrIM Multiphoton microscope, mounted ventrally. Ti:Sapphire laser excited GFP using 860 nm and mCherry with 1100 nm pulsed light through a 25X/1.2NA LWD water-dipping Plan Fluor objective. (**C** and **D**) The anterior mesenteric artery (AMA) is shown budding off the dorsal aorta (DA), with main glomerular arterioles (GA) draining blood through the glomerulus (G) and filtering into the tubules (T). Images are shown (C) merged and (D) α*-*Sma signal as maximum intensity projections. Scale bars represent 20 μm. (**E** and **F**) These datasets were rendered in AMIRA to visualize how the vessels were orientated in 3D. (**G**) Schematic representing the flow of blood (blue arrows) through the glomerular vasculature. Yellow cells represent renin-expressing cells at the AMA location, red cells represent α-Sma-positive cells and green represents the *wt1b*-GFP-positive glomerulus.

The pronephros is the first kidney to form across all vertebrates [56]. Although it lacks the ability to filter blood in higher vertebrates, the pronephros is the first functional blood filtration organ to form in fish and amphibians [57,58]. The zebrafish pronephros is a simple structure consisting of two tubules fused at the midline, ventral to the dorsal aorta and develops rapidly between 3 and 5 days post-fertilization (dpf) [59] (see Figure 1). The pronephric tubular segmentation shares similarities with kidneys of more complex vertebrates and the expression of genes that pattern these have been shown to be highly conserved [60].

**Figure 2:**
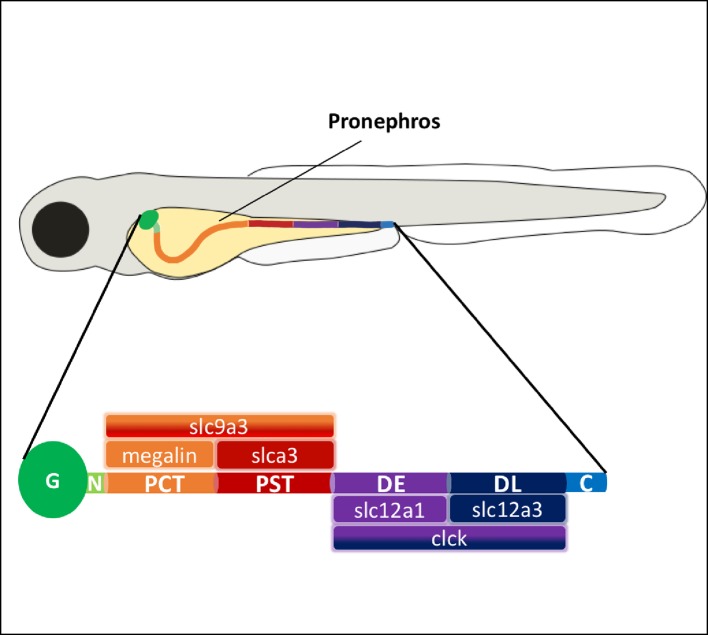
Graphical representation of pronephric zebrafish kidney (∼4dpf; straightened) (G) Glomerulus, (N) Neck, (PCT) Proximal Convoluted Tubule, (PST) Proximal Straight Tubule, (DE) Distal Early, (DL) Distal Late, (C) Cloaca. Specific segments of the pronephric kidney show conservation of mammalian tubular kidney markers.

The first cells destined for pronephric development originate from the intermediate mesoderm that gives rise to the kidney and blood [61] and express renal markers including pax2a, pax8 and lhx1a [62,63]. The zebrafish pronephric tubule was initially thought to be a simple structure, but distinct cell types are now known to pattern the tubule [61]. Eight distinct regions have been identified in the zebrafish pronephric tubule compared with nine distinct regions in the mammalian tubule [60,64]. Each region is defined by specialized cells and transporters aiding in the reabsorption of ions and nutrients [65]. The long stretch of tubular epithelium of the pronephros is subdivided into two proximal segments, two distal segments and a short duct segment (see Figure 2). Similar to the mammalian metanephros, the zebrafish pronephros contains a short neck segment that connects the tubule to the glomerulus and expresses rfx2, a marker for ciliated cells [66]. The proximal segment of the zebrafish kidney is defined by the expression of slc9a3, which is also seen in mammals [67]. Slc9a3 encodes for the epithelial brush border sodium hydrogen exchanger, which contributes to pH balance and promotes survival [68]. The proximal tubule of the pronephros is further subdivided into a convoluted and straight tubule. The proximal convoluted tubule (PCT) expresses the functionally conserved megalin [69], whereas the proximal straight tubule (PST) expresses both slc9a3 and slc13a3, suggesting the presence of multi-ciliated transporting cells [60,64]. A major difference in the kidney structure between freshwater fish and mammals is the tubule arrangement following the proximal segments. The mammalian tubule contains the loop of Henle, which functions predominantly for water reabsorption – a feature that is not required in freshwater fish. The distal segments comprising of the distal early (DE), distal late (DL) and pronephric duct (PD), all express the protein clck [60]. Interestingly, the DE segment in the zebrafish pronephros specifically expresses slc12a1, which in mammals is restricted to the thick ascending loop (TAL), and the DL segment expresses slc12a3, suggesting that the zebrafish pronephric DE and DL segments are analogous to the mammalian TAL [64]. To define the specific segments and the genes expressed within each segment, Wingert and Davidson [60] used a functional genomic strategy to isolate and localize markers of different renal cell types in the zebrafish pronephros. Despite gene conservation showing many similarities between the simple pronephric tubule and the mammalian metanephros, the latter has developed a highly complex collecting duct system into which the tubules drain. In the pronephros, the excretion system is simplified with the two tubules coming together at the pronephric duct and draining directly into the cloaca.

## The zebrafish mesonephros

Mesonephric kidney development was elegantly mapped using *podocin-*mCherry *and cadherin-17-*GFP transgenic reporter fish to differentially mark podocytes and tubular cells [70]. As development continues, thickening and convolution of the pronephric duct was followed by the appearance of new mesonephric podocytes at approximately 12 dpf, which marks the onset of mesonephric kidney development [70]. A pair of newly developing glomeruli appear on either side of the pronephric duct by 14 dpf and the newly forming nephrons fuse to the distal regions of the pronephros, which appears to act as a scaffold [62]. Nephrogenesis continues in an anterior to posterior direction in the trunk region but by 20 dpf also occurs in the rostral (head kidney) region with most nephrogenic events taking place within this region [70]. Nephrons in the trunk region of the kidney have been shown to possess secondary branching tubules, comparable to tubules seen in the metanephric mammalian kidney [35].

**Figure 3:**
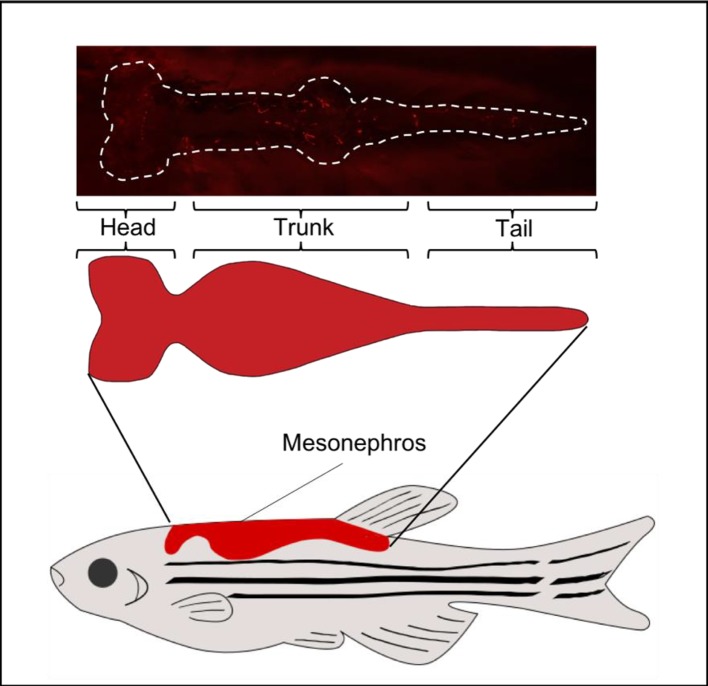
Graphical representation of adult zebrafish mesonephric kidney The mesonephric kidney is segmented into the head, trunk and tail (shown in red) together with a ventral view of the adult kidney in *Tg*(*ren:LifeAct-RFP*) fish showing comparative expression of *ren*:LifeAct-RFP in the head, trunk and tail regions of the kidney.

As the zebrafish reaches sexual maturity, at approximately 3 months post-fertilization, expression of both nephrogenic and podocyte markers decreases [61]. The adult zebrafish mesonephros (see Figure 3) contains upwards of 150 nephrons – the actual number directly correlating to the body mass of the fish – and nephrogenesis continues at a low level, throughout adult life, in order to replenish damaged nephrons and maintain a stable mesonephric nephron count [70,71]. In the event of kidney injury, for example following gentamycin injections, it has been shown that the kidney starts to regenerate within 48 h. Nephrogenesis reaches maximal levels at around 14 days post-injury, and repair follows the conventional nephrogenic mechanism [70]. Hence the zebrafish is well suited as a model for studying kidney development and disease, even though it lacks the complexity of the metanephros. Investigation of kidney injury and repair in the mesonephros are possible due to its *de novo* regenerating capability.

## Transgenic zebrafish

The ease of generating transgenic zebrafish has allowed the establishment of a vast number of tissue-specific transgenic reporter lines. Most commonly transgenic lines utilize a promoter for the gene of interest driving the expression of a fluorescent reporter. Several methods for generating transgenic fish have been described; however, recently the use of transposons has increased the efficiency. Transposons are genetic elements that are able to move between chromosomal loci throughout the genome [72]. Co-injection of transposase mRNA together with the transgenic construct bearing tol2 transposon recognition sites activates a ‘cut and paste’ mechanism for the insertion of desired DNA into the genome [72]. There is no control over the number of insertion sites generated or their location in the genome; however, the high fecundity of the zebrafish means that large numbers of eggs can be injected and larvae carrying the transgene can be selected by monitoring fluorescence, typically early in development.

To understand the molecular and cellular mechanisms underlying zebrafish kidney development, several different lines have been generated including *Tg(pod:mcherry), Tg(wt1b:GFP*) and *Tg(*cdh17:GFP) transgenic fish, which have been used to follow mesonephros development [70]. Below we have summarized and selected strains of transgenic zebrafish lines that are commonly used for studying kidney development and function.

### ren:LifeAct-RFP

**Figure 4:**
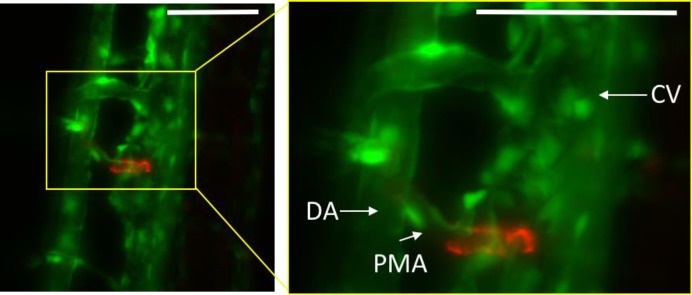
Renin expression at 5 dpf *Tg(ren:KillerRed^mem^*;*kdrl:GF*P) larvae were anaesthetized, embedded in 0.5% agar and mounted on a home built SPIM system [104], head down and imaged ventrally using single laser illumination through a 16X/0.8NA water LWD Plan Fluor objective. *ren*:KillerRed^mem^ expression was excited at 561 nm, *kdrl*:GFP signal at 488 nm. The posterior mesenteric artery (PMA) buds off the dorsal aorta (DA) and caudal vein (CV). Renin expression is shown extending along the PMA at this time point. Images are maximum intensity projections. Scale bars represent 50 μm.

Although the renin gene is highly conserved across mammals, the renin promoter sequence in zebrafish is less well conserved [46]. However, mammalian renin transcription is regulated by the binding of cAMP and RBP-J [36,73] and the 6.4 kb zebrafish promoter includes recognition sites for both of these transcription factors. Transgenic zebrafish, in which the 6.4 kb renin promoter drives the expression of red fluorescent protein (LifeAct-RFP), was used to identify renin expression in the larval kidney and expression correlated directly with data from renin *in situ* hybridization experiments that showed renin expression as early as 24 hpf [74]. Although renin is expressed from 24 hpf, the pronephros does not show active filtration until 72 hpf suggesting that renin might have a role in kidney development rather than ion homeostasis at this early stage [58,61,74]. The requirement of a functional RAS in kidney development has previously been suggested in rodents since all RAS components are expressed early in rat gestation [75]. In common with mammalian JG cells, zebrafish renin-expressing cells co-express smooth muscle and pericyte markers from ∼96 hpf [76,77]. In the zebrafish pronephros, these cells are present in a post-glomerular position along the anterior mesenteric artery (AMA) [74,78–80] and from 5 dpf at the posterior mesenteric artery (PMA, see Figure 4) [74]. Taken together with the low blood pressure system in zebrafish, it suggests that tubulo-glomerular feedback is not present in the pronephric kidney [74].

The *ren:LifeAct-RFP* transgene indicated the presence of renin-expressing cells at angiogenic sprouts, analogous to reports of renin-expressing cells at the tips of sprouting vessels in rats [81] and may suggest a function similar to that described in murine kidney vasculature [35]. The use of axitinib to inhibit angiogenesis in *Tg*(*ren:LifeAct-RFP*) fish prevented the formation of the AMA and revealed the expression of renin at the vessel tip [74]. Later in zebrafish development, renin-expressing cells are located mid-vessel, as is seen in the developing mammalian arterial tree [82]. However, in the zebrafish mesonephros, which continually undergoes nephrogenesis, no renin-expressing cells were observed on the newly forming vessels. This differs from their presence during the development of AMA and suggests distinct functions for the renin-expressing cells.

In mammals, recruitment of renin-producing cells is regulated by nitric oxide derived from neighbouring endothelial cells [83]. The positioning of renin-expressing cells along the afferent arterioles is also influenced by connexin-40 gap junctions, which exist between renin-expressing smooth muscle cells and endothelial cells [84,85]. The requirement of endothelial cells for the maintenance of renin-expressing cells in zebrafish was demonstrated by a double transgenic zebrafish line resulting from a cross between *Tg(ren:LifeAct-RFP*) and the cloche mutant (*clo*^m39^), which lacks endothelial cells. This confirmed distinct lineages for renin-expressing cells and endothelial cells. Although renin was initially expressed in developing *Tg(ren:LifeAct-RFP*;*clo*^m39^**) larvae, all renin expression was lost by 96 hpf [74].

The *Tg(ren:LifeAct-RFP*) transgenic line also enabled the characterization of granulated renin cells in the zebrafish mesonephros. Two distinct renin-expressing perivascular cell types were identified. Epithelioid-like cuboidal renin-expressing cells along the afferent arterioles contained granules suggestive of renin activation and secretion, whilst flat non-granulated cells enveloped the efferent arterioles. This raises questions regarding functional differences between cells having distinct morphologies. In mice, a subset of renin cells along the efferent arterioles are granulated [7,86]. The appearance of granules and pronounced endoplasmic reticulum and Golgi apparatus, all required for renin processing, is dependent, in mice, on the Notch pathway [36]. The zebrafish mind bomb mutant *mib*^ta52b^ lacks the ubiquitin ligase mind bomb, which ultimately impairs activation of the Notch receptor [87]. The need for an active Notch pathway for zebrafish renin cells to be expressed was demonstrated using this mutant. Zebrafish *mib*^ta52b^ failed to develop renin cells as demonstrated by a lack of renin expression along the anterior mesenteric artery [74].

### wt1b:GFP

The Wilms’ tumour gene WT1 encodes a zinc finger transcription factor and is a major regulator of mesenchymal progenitors in many organs including the heart, kidney, spleen and gonads [78]. Dysregulation of WT1 is linked to a paediatric renal cancer and leads to abnormalities in the urogenital tract, suggesting that WT1 is essential for proper kidney development and the health and maintenance of a functioning glomerulus [88,89]. Mice lacking *Wt1* also lack ureteric buds, and though the meta-nephrogenic blastema is formed, apoptosis soon follows suggesting that Wt1 signalling is required for the mesenchyme blastema to form ureteric buds [90].

Zebrafish contain two homologues of the WT1 gene termed *wt1a* and *wt1b* [91]. The genes appear to have different roles since a morpholino knockdown of *wt1a* induces oedema, whereas a lack of *wt1b* results in subtle oedema coupled with body curvature [92,93]. The presence of oedema suggests an inability to regulate fluid homeostasis. Both *wt1* genes are highly expressed in the developing kidney [91] but expression diminishes in adulthood. *Tg(wt1*) transgenic zebrafish were generated in which GFP was driven by the *wt1b* promoter that was delineated by sequence conservation with the *Wt1* sequence previously described in mice [92]. A GFP signal was observed from 35 hpf, caudal to the second somite pair – the area giving rise to glomeruli, the developing pronephric tubules and the proximal tubules. The transgenic line showed that fish with inactivated Wt1a lacked glomeruli whereas lack of Wt1b resulted in the formation of renal cysts, similar to the deregulatory effect of WT1 in humans. The *wt1* line recapitulates the *wt1* expression during kidney development and presents an ideal tool for studying kidney development *in vivo*.

### α-sma:GFP

The smooth muscle actin promoter alpha (α-*sma*), fused either to a fluorescent GFP or m-cherry reporter, has been used as a marker of vascular smooth muscle cells and pericytes, in developing zebrafish [80]. At 3 dpf, there is little expression of α-*sma* in the kidney, though faint expression can be seen within renin-expressing cells [96]. By 4 dpf, there is more extensive α-*sma* expression within the glomerular arterioles, as can be seen using multiphoton microscopy (Figure 1C,D). α-*sma*:reporter fish were used to mark co-localization of renin with a subset of mural cells [74].

### fli1/kdrl:EGFP

The promotors for endothelial cell-specific genes such as *fli1* and *flk-1/kdrl* have been used to drive the expression of fluorescent reporters in the embryonic vasculature of developing zebrafish and have been used successfully to demonstrate the distinction between renin-expressing cells and the endothelium [74].

## Drug-induced nephrotoxicity in the Zebrafish

A number of drugs are known to cause kidney damage, including gentamicin and puromycin. Gentamicin was used for the induction of podocyte ablation, glomerular injury and the study of subsequent nephron regeneration. After demonstrating that Wt1b is a developmental marker of mesonephric nephrons, Zhou et al. [70] showed a dramatic increase in the expression of Wt1b up to 14 days post-injury. Rider et al. [76] used puromycin to effect podocyte injury, which was validated by demonstrating podocyte effacement. They also developed an assay for glomerular barrier function to demonstrate the loss of discriminative filtration. The dynamics of abnormal protein excretion was monitored using semi high-throughput 70 kDa dextran excretion and was evident 24-h post injury.

The bacterial nitro-reductase (NTR) system is commonly used for the ablation of cells by treatment with the prodrug, metronidazole. Metronidazole (MET) is converted to a DNA cross-linking cytotoxin in cells expressing NTR under a tissue-specific promoter, ultimately causing cell type-specific apoptosis. This system has also been used to target podocytes and led to the development of a functional assay for glomerular barrier formation using fluorescently labelled protein [94], and identified factors involved in the process of podocyte injury [95]. The NTR system has been repeatedly shown to function effectively; however, it has distinct disadvantages: it is slow acting, takes between 24 and 72 h to induce apoptosis, and the MET prodrug can cause off-target toxicity if not washed out [96]. This limits the studies that can be performed, particularly if the events being investigated occur over short timescales.

### ren:KillerRed_mem_

The optical transparency of the zebrafish larvae can be utilized to enable optogenetic rather than pharmacological approaches to perform targeted ablation. This is achieved using photosensitizing proteins such as KillerRed [97,98]. These proteins produce large amounts of reactive oxygen species upon illumination with a specific wavelength of light and are routinely used in photodynamic therapy [99,100]. Upon light-stimulated excitation of electrons in the chromophore region of photosensitizing proteins, the excited electrons interact with molecular oxygen, generating free radical superoxide anions – a process known as phototoxicity. Normal fluorescent proteins are relatively shielded from this effect due to their protein conformation; the β-barrel shape of the protein stops too much molecular oxygen from reaching the central fluorescence-generating region. KillerRed differs from these proteins in that it has a water channel linking the intracellular solvent to the protein’s methylene bridge, facilitating transport of molecular oxygen to the excited electrons [101]. Subsequently, as photobleaching and phototoxicity occur in KillerRed-expressing cells, much larger amounts of reactive oxygen species are produced than in normal fluorescent proteins, causing oxidative damage and leading to apoptosis in KillerRed-expressing cells.

This technology has been applied successfully in cell culture systems [98], mouse models [102] and zebrafish [103]; however, these studies illuminated a large number of cells using either epifluorescence or confocal light. By targeting the illumination, specific cells can be very accurately killed without having to use pharmacological intervention or an extremely high laser power. Using this principle, a strain of fish was generated, *Tg(ren:KillerRed _mem_*), in which a membrane-bound form of KillerRed was expressed under the renin promoter described above [104]. As in the *Tg(ren:LifeAct-RFP*) fish, renin expression was first seen at 2.5 dpf at the AMA location, and from approximately 5 dpf at the PMA location (see Figure 4) [74].

Using these fish, KillerRed-expressing cells were targeted with laser light (at 561 nm) using single plane illumination microscopy (SPIM). Incident light is in the form of a plane (light sheet) and emitted light is collected through a separate objective lying perpendicular to the illumination objective, confining axial resolution to the width of the light sheet and minimizing off-target photobleaching [105]. This allowed rapid (within 1 h) simultaneous targeted ablation of the group of cells at the AMA in 3 dpf *Tg(ren:KillerRed _mem_*) fish. By further refining the illumination through the integration of a Bessel beam into the imaging arm of the microscope, specific individual cells were rapidly and reproducibly targeted within 30 min [104]. The speed and precision of this technique will make it invaluable as a tool for studying rapidly occurring processes in the zebrafish kidney, particularly if single or small groups of cells are to be targeted.

## Challenging Zebrafish RAS

### RAS inhibitors

Since the RAS has been linked to hypertension and chronic kidney disease, there has been much interest in developing pharmacological inhibitors to attenuate or block its effects including angiotensin receptor blockers and angiotensin-converting enzyme inhibitors. Captopril, an ACE inhibitor, has been shown to decrease plasma AngII, elevate levels of plasma AngI and consequently lead to an increase of renin activity. Captopril is a highly effective inhibitor of ACE in zebrafish, and it was shown that captopril treatment over the course of 7 days elevated renin mRNA levels and the fish were unable to survive in low salt water indicative of a lack of osmoregulation [74].

Renin inhibitors have been developed, and cause a decrease in plasma renin activity, but were largely discontinued due to their short duration of action, and issues regarding activity and bioavailability [106]. Aliskiren is currently the only direct renin inhibitor available that is capable of preventing the synthesis of all angiotensins [26]; however there are no reports, to date, suggesting that Aliskiren is active in zebrafish.

Efforts are currently underway to establish an assay for the direct measurement of zebrafish angiotensins in the plasma using mass spectrometry, a well-documented method for the analysis of angiotensins; however, there are no reports of its application in lower vertebrates. The amino acid sequence for teleost angiotensinogen is known [107], and this enabled the chemical synthesis of the predicted peptides for zebrafish AngI and AngII using solid phase peptide synthesis and their use for the development of a robust assay for the accurate detection of AngI and AngII in zebrafish (Hoffman, unpublished).

### Salinity

Several endocrine systems are shown to be involved in ion uptake in fish including prolactin and cortisol [51,108]. The presence of multiple regulators accentuates the importance of ion homeostasis control in fish. In mammals, the main regulator of salt uptake and balance is AngII [14]. Previous studies have investigated the role of renin and the RAS in zebrafish when exposed to homeostatic threats such as low salinity [51]. An increase in mRNA levels was observed when fish were exposed to a salt concentration 1/20th lower than that of regular water [49,74]. An opposite effect was seen when the fish were exposed to 100 times higher salt concentration. Although changes in renin were observed the levels did not correspond to the changes of prolactin [51]. In order to further investigate the role of the RAS in zebrafish, Kumai et al*.* [50] examined the ion regulatory effects of AngII. A decrease in sodium reabsorption was demonstrated following renin knockdown using a renin morpholino. On the other hand, sodium reabsorption increased following exposure of the zebrafish to extracted AngII. These observations suggest a strong involvement of AngII in osmoregulation; however, some caution in the interpretation of the effect of morpholinos is needed since they are known to induce toxic side effects.

## Conclusions

Given the conservation of RAS genes between species and functions of the pronephric and mesonephric kidney, the zebrafish is proving to be a very useful organism for asking basic questions about the involvement of RAS components in kidney development, disease and regeneration, for example, by studying the cellular function of granulated and non-granulated renin cells at the JGA, or the impact of RAS inhibition at different stages of renal injury.

The role of renin in the developing kidney versus the regenerating kidney could be investigated through the specific ablation of renin cells at vessel sprout tips during angiogenesis versus juxtaglomerular cells during nephrogenesis. Ablation studies and fluorescently marked renin cells may also shed light on how the new vasculature is integrated with new nephrons.

The number of transgenic tools, injury models and potentially high-throughput assays continues to expand, making the zebrafish a key player in kidney research and the identification of new therapeutic strategies. With the development of genome editing technology, gene knockout in the zebrafish is now achievable, and this opens the door to the development of new informative strains and tools, which will greatly enhance interrogation of the RAS in zebrafish.

## Abbreviations

ACE (1 or 2): angiotensin converting enzyme type 1 or 2
AMA: anterior mesenteric artery
Ang (I or II): angiotensin type 1 or 2
At (1 or 2): angiotensin receptor type 1 or 2
C: cloaca
CV: caudal vein
DA: dorsal aorta
DE: distal early
DL: distal late
dpf: days post fertilization
G: glomerulus
GA: glomerular arterioles
hpf: hours post fertilization
JG(A): juxtaglomerular (apparatus)
MET: metronidazole
NTR: nitro reductase
PCT: proximal convoluted tubule
PD: pronephric duct
PMA: posterior mesenteric artery
PST: proximal straight tubule
RAS: renin–angiotensin system
SPIM: selective plane illumination microscope
T: tubules
TAL: thick ascending loop
